# Hematology, Biochemistry Reference Intervals, and Morphological Description of Peripheral Blood Cells for a Captive Population of *Crocodylus intermedius* in Colombia

**DOI:** 10.3389/fvets.2021.694354

**Published:** 2021-08-26

**Authors:** Steven Barajas-Valero, Cristian Rodríguez-Almonacid, Zulma Rojas-Sereno, Carlos Moreno-Torres, Nubia E. Matta

**Affiliations:** ^1^Grupo Caracterización Genética e Inmunología, Departamento de Biología, Universidad Nacional de Colombia, Bogotá, Colombia; ^2^Departamento de Salud Animal, Facultad de Medicina Veterinaria y de Zootecnia; Universidad Nacional de Colombia, Bogotá, Colombia; ^3^Centro de Investigación para la Sustentabilidad, Facultad de Ciencias de la Vida, Universidad Andrés Bello, Santiago, Chile

**Keywords:** captive orinoco crocodile, *Crocodylus intermedius*, reference intervals, hematology, RBC, Colombia

## Abstract

The Orinoco crocodile (*Crocodylus intermedius*, Graves, 1918) is the most threatened crocodilian of South America. There is only scarce information available about the physiology of this neotropical crocodile. This study aimed to propose baseline hematological and biochemistry reference data and intervals and a morphological description of the peripheral blood cells of captive *C. intermedius*. Blood was collected from 318 clinically healthy individuals maintained in captivity at Villavicencio, Colombia. Eight of these individuals were sampled and resampled, and these data were compared. Reference intervals were proposed for hematological values [packed cell volume (PCV), red blood cell count, white blood cell count, mean corpuscular volume, mean corpuscular hemoglobin, mean corpuscular hemoglobin concentration, hemoglobin, and white blood cell count differential counts] and biochemistries [total solids, alanine aminotransferase (ALT), aspartate aminotransferase, alkaline phosphatase, lactate dehydrogenase, creatine kinase, glucose, albumin, cholesterol, uric acid, creatinine, and lactate] including adults and juveniles, males and females' crocodiles. Blood cell morphology for the species is described. Significant differences between sex and age were observed. The intraindividual analysis concluded differences for total solids (*P* ≤ 0.01) and red blood cell counts (*P* ≤ 0.01). Some biochemical analytes showed a moderate correlation between them, such as ALT–alkaline phosphatase and ALT–uric acid. We present here novel and baseline data with special importance for the clinical diagnosis, improving the national reintroduction programs from either *in situ* and *ex situ* populations.

## Introduction

The Orinoco crocodile (*Crocodylus intermedius*) is distributed in the Orinoquean basin in the wetlands of Colombia and Venezuela and is one of the six crocodilian species found in Colombia (1, 2). This species is the most threatened neotropical crocodilian due to historical overexploitation and other anthropic interventions (3–5). It is classified as critically endangered in the International Union for Conservation of Nature's endangered species categories and in Appendix I by Convention on International Trade in Endangered Species of Wild Fauna and Flora (6, 7).

The national *ex situ* conservation program has been partially successful with reintroduction into various Orinoquean watersheds (6, 8, 9). However, research on *C. intermedius* has focused primarily on ecology and population status with few veterinary health investigations for the species in Colombia (10–12).

Veterinary diagnostics for crocodilian species rely mainly on postmortem examination, although they are globally maintained in controlled facilities for conservation and other purposes (13). Because reptiles and especially crocodilians do not exhibit early signs of discomfort or disease, routine observations are not practical or neither recommended. However, blood testing could offer more information and be performed twice per year for preventive medical programs (14, 15).

This leads to a frequent and tedious issue with reptiles; the lack of baseline information limits the application of diagnostic tools (16). The establishment of hematological and cytological reference data and intervals is essential for obtaining baseline criteria for a health assessment (17). Such testing is useful for monitoring of reintroductions and immunological and/or environmental toxicology studies (18–20).

This study aimed to propose baseline reference data and reference intervals for hematology and serum biochemistry and provide a peripheral blood cell description for captive *C. intermedius* in Colombia.

## Methods and Materials

### Ethical Statements

The Science Faculty ethics committee of the Universidad Nacional de Colombia approved the methodology for this research through N° 03-2019.

### Population

Blood samples from 318 clinically healthy *C. intermedius* were collected during two periods that cover 6 years (2010–2013 and 2019–2020). All individuals were held in captivity at the Estación Biológica Tropical Roberto Franco of the Universidad Nacional de Colombia, located in Villavicencio, Meta, Colombia (latitude 4.13°, longitude 73.63°), at 419 meters above mean sea level. Local temperature oscillates between 20 and 32°C with a mean relative humidity of 76%, an average annual rainfall of 4.008 mm, and a unimodal rainfall regime. Sex and age were recorded for all individuals. Juvenile and subadults were sampled during the first phase; only subadults were sampled in the second phase. Individuals did not receive any medical treatment or intervention 3 months before sampling. Morphometry and weight data were documented for 41 individuals in the second phase. Eight individuals were sampled twice during the 6-year period.

### Sampling

Individuals were captured and physically restrained (21). To diminish lymphatic hemodilution possibility, blood was obtained from the ventral coccygeal vein using a 5-ml syringe without anticoagulant, with a 21 G^*^1½ -inch needle. Immediately after sample obtention, four thin blood smears per individual were prepared with fresh non-heparinized blood using the slide-to-slide technique. Blood smears were dried using low airflow at environmental temperature and fixed with absolute methanol for 5 min. Whole blood was placed in sodium heparin vials (Liquemine, Roche) and carefully mixed. These vials were stored at 4°C and processed within the next 8 h after sampling. Serum was obtained using separating gel vials (Liu yang Sanli Medical Technology Development Co Ltd., China), centrifugated at 4,000 rpm per 10 min, and then stored at −20°C for subsequent analysis.

### Hematology

Blood smears were stained using Wright and Giemsa. Microscopic evaluation was carried out with an Olympus CX41 microscope (Olympus Corp., Tokyo, Japan). Red blood cell counts (RBC) and white blood cell counts (WBC) were performed manually with Neubauer chamber and Natt–Herrick solution [1:100 dilution; ((22), p. 735–738)]. The corner squares and central one (0.4 mm each) in the center (1 mm) of the Neubauer chamber were considered for RBC. Differential leukocyte counts were performed on Wright-stained blood smears based on 100 counted leukocytes in the 100× objective (23–32). The PCV was determined using the microhematocrit method [5 min at 12,000 rpm; ((33), p. 131–141)]. The hemoglobin (HGB) concentration was measured with spectrophotometry using the BTS-350 equipment (Biosystem, Spain). Mean corpuscular volume, mean corpuscular HGB, and mean corpuscular HGB concentration (MCHC) indices were calculated following Eatwell et al. (23).

### Serum Biochemistry Analyses

Total solids (TSs) were determined using hand refractometry (Scientific, China). Serum biochemistry analyses were performed by spectrophotometry using Biosystem BTS-350 (Biosystem, Spain) and Spinreact kits (Spinreact, Spain). Measured analytes included glucose, aspartate aminotransferase (AST), alanine aminotransferase (ALT), albumin, alkaline phosphatase (ALP), cholesterol, uric acid (UA), creatinine (CREA), creatine kinase (CK), lactate, and lactate dehydrogenase (LDH).

### Intraindividual Analysis

To determine if there were differences between hematological and serum biochemistry parameters within the same individual in juvenile and adult stages (5-year interval), intraindividual comparisons were made among PCV, TS, mean corpuscular volume, ALT, RBC's, and WBC's relative and absolute counts.

### Peripheral Blood Cell Characterization

Digital microphotographs of blood cells were obtained at 100× objective using an Olympus DP27 digital camera and processed with cellSens Standard 1.13 software (Olympus, Tokyo, Japan). Measurements were processed with ImageJ® software [(34), p. 671–675] for mature erythrocytes, polychromatophils, thrombocytes, heterophiles, lymphocytes, eosinophils, monocytes, and basophils.

### Statistical Analysis

Reference intervals (RIs) for hematology and serum biochemistry data were generated in accordance with the American Society of Veterinary Clinical Pathology guidelines (35). To verify data distribution, Shapiro–Wilk and Kolmogorov–Smirnov with Lilliefors correction tests were applied according to the sample size. Outlier detection was performed through the Tukey test with the Carling modification and robust kernel-based local outlier detection index. The values 2.5 and 97.5% were considered as lower and upper limits for the RI, respectively. Ninety percent confidence intervals for each one of the limits were calculated. The Mann–Whitney test for independent samples was implemented for sex and age (juvenile and adult) group comparison.

Intraindividual comparisons were developed by Wilcoxon paired samples testing for non-parametric data or a T-paired test for parametric data. Correlation analyses were made between the following paired analytes: CK–AST, CK–LDH, CREA–UA, ALT–LDH, ALT–ALP, ALT–UA, and ALT–cholesterol, using Spearman's rho or Pearson tests. Simple and multiple linear regression for hematology and biochemistries vs. weight and total length morphometrics were explored. All analyses were carried out with RStudio® (v4.0.0) software. Statistical significance for all analyses was set at *P* < 0.05.

## Results

### Population

A total of 318 clinically healthy individuals (249 adults and 69 juveniles; 254 of them females, 52 males, and 12 not sex-identified) were sampled. Bodyweight (50.14 ± 10.6 kg) and total length (224.03 ± 20 cm) showed a direct proportional correlation (*P* = ≤ 0.01, rho = 0.77; Supplementary Figure 1).

### Hematology and Serum Biochemistry Analyses

Hematological and serum biochemistry RIs for all *C. intermedius* were established (Table 1). Heterophils were the most frequent leukocyte (33–81%), followed by lymphocytes (up to 50%) and basophils (till 16%). Significant differences between sexes were observed, where females had higher values for PCV (*P* ≤ 0.01), CK (*P* = 0.03), MCHC (*P* = 0.04), HGB (*P* ≤ 0.01), ALP (*P* = 0.03), CREA (*P* = 0.01), RBC (*P* = 0.04) and WBC (*P* = 0.01; Supplementary Table 1). Significant differences between ages for TS (*P* ≤ 0.01) and RBC (*P* = 0.02; Supplementary Table 1) were observed, where adults had higher values for both analytes. Most of those differences were within the established ranges in this study; groups with substantial differences were presented separately (Table 2).

**Table 1:** Hematological and biochemical reference values and intervals (90% CI) for captive *Crocodylus intermedius*.

**Analyte**	**Mean**	**SD**	**LL (90% CI)**	**UL (90% CI)**	**Min–Max**	***n***	***p*-value**
PCV (%)	26.3	3.7	19.0 (18–19)	33.0 (32–34)	17–36	314	<0.01
TS (g/dl)	7.0	1.4	6.4 (4–4.4)	9.8 (9.6–10)	3.9–10.2	318	<0.01
HGB (g/dl)	8.2	1.2	5.8 (5.2–6)	10.5 (10–11)	5.1–12.1	117	<0.01
RBC (10^6^/μl)	1.1	0.4	0.6 (0.57–0.66)	2.1 (2.02–2.19)	0.47–2.28	269	0
WBC (10^3^/μl)	6.8	2.5	3.1 (2.6–3.1)	11.4 (10.9–11.6)	2.2–15.1	307	0.03
MCV (fl)	263	76.7	134 (115–139)	417 (404–455)	100–592	273	0.01
MCH (pg)	10.1	2.5	6.8 (5.7–6.9)	16.4 (14.3–17.4)	5.7–17.4	84	0.03
MCHC (g/dl)	33.1	5.4	23.1 (21–24)	42.3 (41–44)	22.1–50.4	114	0.73^c^
H (%)	60.8	12.1	33.0 (28–34)	81.0 (80–82)	20–83	301	<0.01
H (cel/μl)	4,104	1,947.1	980 (866–1,066)	8,406 (7,992–9,013)	300–9,595	309	0.01
L (%)	27.2	10.5	11.0 (9–12)	50.0 (48–52)	6–65	301	<0.01
L (cel/μl)	1,721	744.4	613 (539–689)	3,424 (3,372–3,501)	304–3,690	304	0
E (%)	3.6	3.3	0 (0–0)	11.0 (10–12)	0–16	307	0
E (cel/μl)	189	150.5	0 (0–0)	694 (488–688)	0–622	301	0
M (%)	1.7	3.4	0 (0–1)	13.0 (11–16)	0–20	276	0
M (cel/μl)	54	85.8	0 (0–0)	277 (266–331)	0–346	275	0
B (%)	6.6	4.2	0 (0–1)	16.0 (16–18)	0–20	304	0
B (cel/μl)	405	245.6	0 (0–60)	979 (899–1,039)	0–1,092	303	<0.01
A^a^ (%)	0.0	0.2	0 (0–0)	3.0 (0–0)	0–3	311	0
A^a^ (cel/μl)	1.8	18.3	0 (0–0)	279.0 (0–0)	0–280	312	0
GLU (mg/dl)	128.1	23.5	81.9 (66–93)	174.2 (164–190)	92–186	39	0.12^c^
ALB (g/dl)	2.0	0.3	1.4 (1.4–1.6)	2.7 (2.6–2.8)	1.4–3	134	<0.01
ALT (u/l)	40.6	21.1	18.9 (14.8–20)	100.0 (90–100)	11.5–100	191	0
AST (u/l)	103	64.6	59.6 (37–66)	354 (306–395)	25–260	134	<0.01
ALP (u/l)	181	79.5	27.3 (25–30)	240 (230–250)	37–400	136	0.54^c^
CHOL (mg/dl)	253	40.3	176 (166–180)	322 (316–342)	164–357	193	0.14^c^
UA (mg/dl)	4.4	1.7	1.2 (0.4–1.7)	7.7 (7.1–8.4)	1.8–7.9	73	0.16^c^
CREA^a^ (mg/dl)	0.5	0.1	0.4 (-)	0.7 (-)	0.4–0.7	127	0
CK (u/l)	1,825	1,288	332 (187–348)	4,789 (4,532–5,915)	187–5,915	89	<0.01
LACT (mg/dl)	66.2	41.8	17.9 (17–18)	127.3 (116–177)	16–178	34	<0.01
LDH^b^ (u/l)	21.5	10.4	-	-	4–47.1	20	0.80^c^

*SD, Standard deviation; LL, Lower limit (percentile 2.5); UL, Upper limit (percentile 97.5); CI, Confidence interval; Min–Max, Minimum and maximum observed values; n, Number of observations; p-value, p-value for normality test. Absent CIs coincide with RIs values. MCV, Mean corpuscular volume; MHC, Mean hemoglobin concentration; MCHC, Mean corpuscular hemoglobin concentration*.

a *90% CI could not be calculated because of data distribution*.

b *RI is not presented due to the small sample size*.

c *Parameters with Gaussian distribution*.

**Table 2:** Hematological and biochemical reference values and intervals for captive *Crocodylus intermedius'* parameters with statistical differences according to sex/age groups.

**Analyte**	**Group**	**Mean**	**SD**	**LL (90% CI)**	**UL (90% CI)**	**Min–Max**	***n***	***p*-value**
TS (g/dl)	Juvenile	6.1	1.1	4 (3.5–4.4)	8.2 (7.8–8.7)	4–8	77	0.06
	Adult	7.3	1.3	4.7 (4.5–5.0)	10 (9.7–10.2	3.9–10.2	241	<0.01
RBC (10^6^/μl)	Juvenile	1.04	0.42	0.7 (0.56–0.83)	2 (1.86–2.13)	0.7–2.0	77	<0.01
	Adult	1.15	0.47	0.6 (0.5–0.69)	2 (1.9–2.1)	0.5–2	200	<0.01
ALP (u/l)	Female	190	85.9	22.2 (0–46.7)	358 (336–386)	37–400	97	0.12^**a**^
	Male	152	49.7	55.2 (25.9–75.4)	250 (229–279)	42–257	35	0.45^**a**^
CK (u/l)	Female	2,629	2,213	312 (187–348)	7,448 (7,235–7,933)	187–8,065	65	0
	Male	1,564	1,224	352 (328–363)	4,946 (2,920–5,915)	328–5,915	27	0.32^**a**^

*SD, Standard deviation; LL, Lower limit; UL, Upper limit; CI, Confidence interval; Min–Max, Minimum and maximum observed values; n, number of observations; p-value, p-value for normality test*.

a *Parameters with Gaussian distribution*.

### Peripheral Blood Cell Characterization

**Figure 1:**
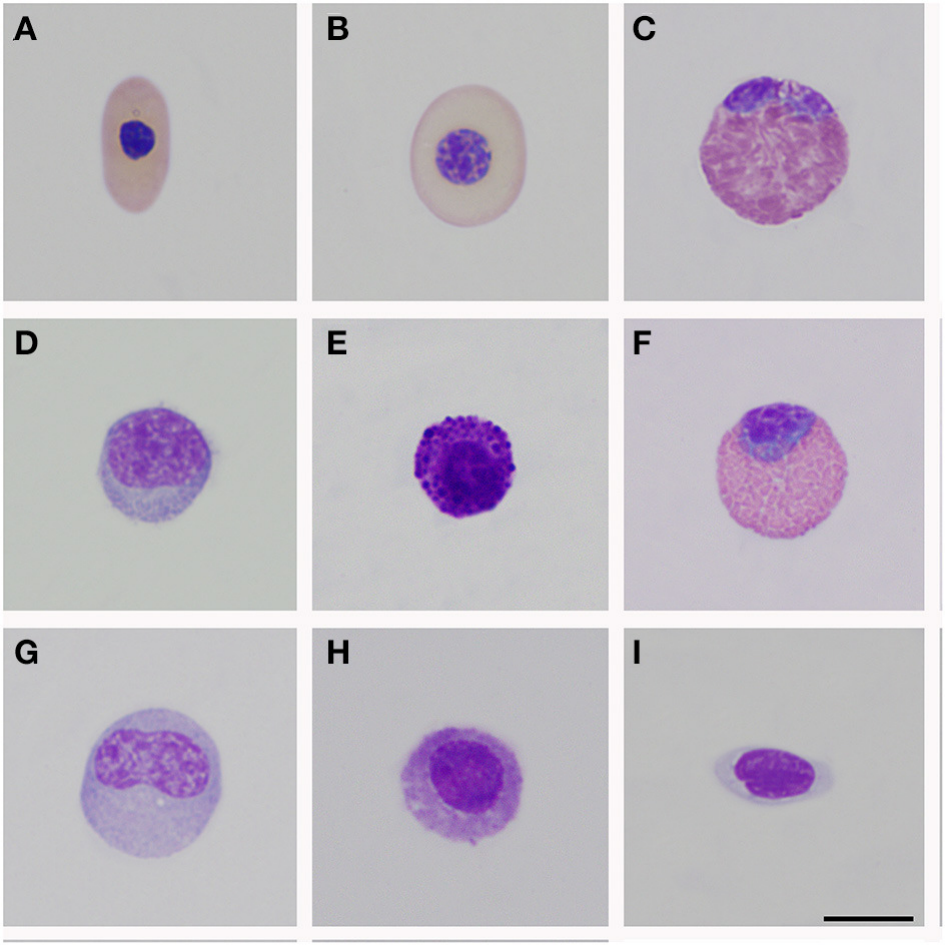
Peripheral blood cells of *Crocodylus intermedius*. **(A)** Mature erythrocyte; **(B)** Polychromatophil; **(C)** Heterophil; **(D)** Mature lymphocyte; **(E)** Basophil; **(F)** Eosinophil; **(G)** Monocyte; **(H)** Azurophil-like; **(I)** Thrombocytes. Bar: 10 μm.

**Figure 2:**
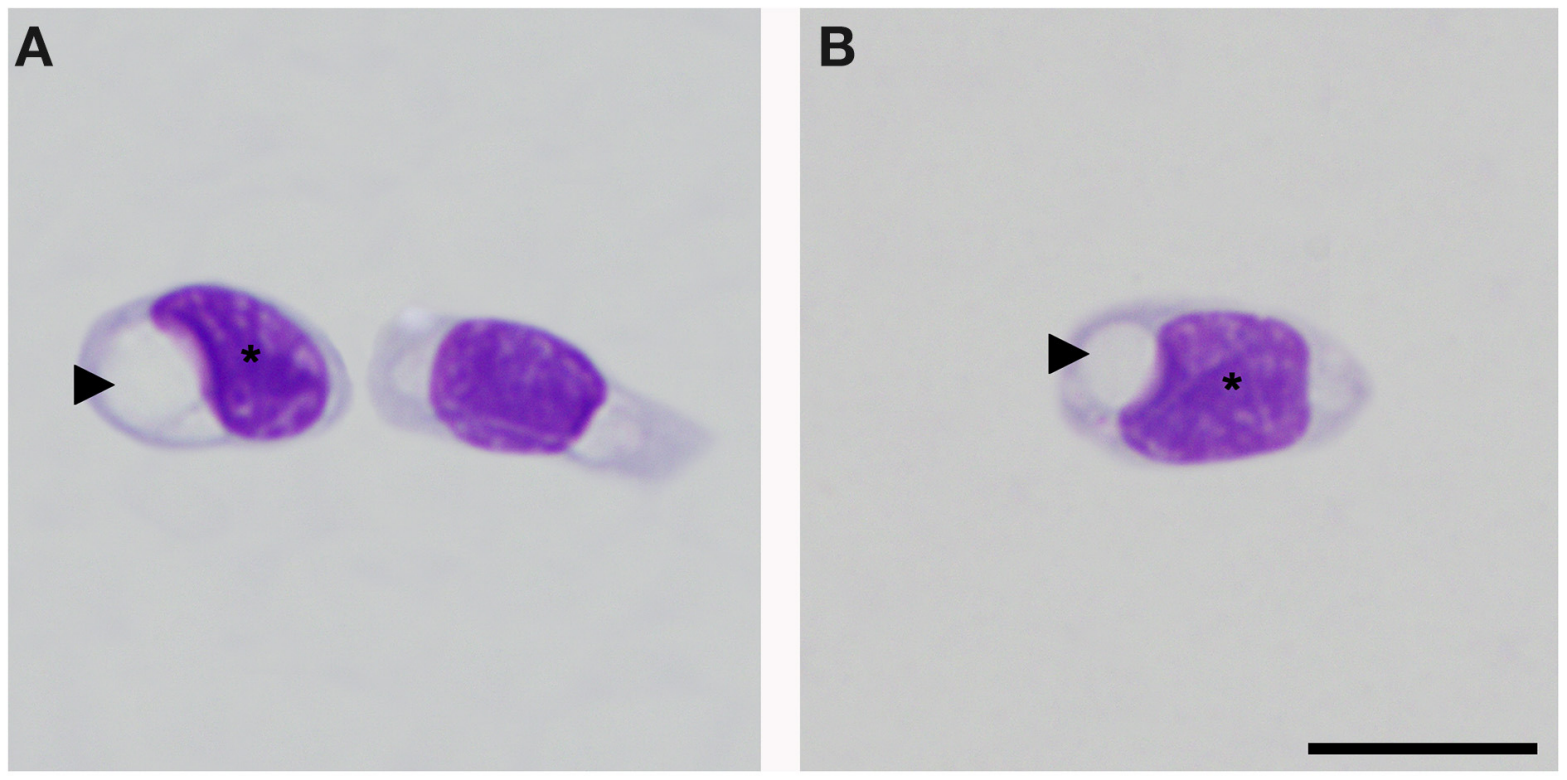
Thrombocytes of *Crocodylus intermedius* that showed an irregular appearance. Cytoplasm with the large colorless vacuole (arrowhead), note the deformed and slightly displaced nucleus (asterisk), and the normal coloration of thrombocyte cytoplasm (right side cell on **A**). Bar: 10 μm.

Morphological description of peripheral blood cells of *C. intermedius* using Giemsa (G) and Wright (W) stains are shown in Table 3 and Figure 1. Morphometric parameters are shown in Table 4. Polychromatophils appeared as larger cells than mature erythrocytes (148.1 ± 8.87 μm *vs*. 108.8 ± 14.4 μm cell area). Azurophils were rarely seen, and they could be easily mistaken or missed in deficient smears (under or overstained). One adult exhibited vacuolated thrombocytes with slight nucleus displacement (Figure 2). All the blood smears were deposited in the GERPH Biological Collection – Biology Department, Universidad Nacional de Colombia.

**Table 3:** Morphology description of *Crocodylus intermedius'* peripheral blood cells on light microscopy.

		**RBC's**		**Granulocyte WBC's**			**Agranulocyte WBC's**		**Thrombocyte**
		**Erythrocyte**	**Polychromatophil**	**Heterophil**	**Eosinophil**	**Basophil**	**Lymphocyte**	**Monocyte**	
**Cytoplasm**	Description	Most frequent cell in peripheral blood. Typically, ellipsoidal shape	Sporadically seen. Round shaped, bigger than mature erythrocytes	Spherical shape. Fusiform granules, overlapping, and poorly differentiated	Scarcely seen. Spherical shape. Contain copious amounts of rounded granules	Scarce to moderate presentation. Spherical shape. Large rounded and copious granules	Spherical, scarce cytoplasm, pseudopodia. Huge size variations	Biggest leukocyte. Plentiful cytoplasm, diverse size vacuoles. Pseudopodia	Elliptic shape, scarce cytoplasm. Clumping commonly observed
	*G*	Pale cream tone	Pale gray	Deep magenta granules, colorless cytoplasm	Eosinophilic granules. Colorless cytoplasm	Dark basophilic granules. Colorless cytoplasm	Deep blue tone	Faint blue-gray tone	Colorless
	*W*	Cream pink	Light cream (yellowish pink)	Frequently colorless					Faint blue-gray tone
**Nucleus**	Description	Centrally located, round shaped and dense chromatin.	Round to amorphous, centrally located, chromatin slightly condensated.	Elliptical or round shaped, peripherally located, usually not lobulated.	Single or bilobated, peripherally located. Could be partially covered by granules.	Centrally located. Partially or totally covered by granules.	Occupying most of the cellular area. Round shaped.	Centrally located, U shaped, chromatin slightly condensed.	Centrally located, slightly oval, chromatin moderately condensed. “Coffee grain” appearance.
	*G*	Dark basophilic	Faint basophilic	Dark basophilic	Light basophilic tone	Dark basophilic	Dark basophilic	Faint basophilic	Faint basophilic
	*W*								

*(G) color acquired on Giemsa stain and Wright stain (W)*.

**Table 4:** Basic morphometric aspects on light microscopy for peripheral blood cells of *Crocodylus intermedius*.

	**RBC's**		**Thrombocyte**	**Granulocyte WBC's**			**Agranulocyte WBC's**	
	**Erythrocyte**	**Polychromatophil**		**Heterophil**	**Eosinophil**	**Basophil**	**Monocyte**	**Lymphocyte**
L	16.1 ± 0.8 μm	16.0 ± 0.6 μm	12.6 ± 1.5 μm	-	-	-	-	-
W	8.2 ± 0.7 μm	11.5 ± 0.5 μm	5.8 ± 0.6 μm	-	-	-	-	-
D	-	-	-	16.6 ± 0.8 μm	14.8 ± 1.4 μm	12.7 ± 1.3 μm	15.4 ± 1.7 μm	10.1 ± 0.7 μm
CA	108.8 ±14.4 μm	148.1 ± 8.9 μm	58.5 ± 1.5	207.9 ± 0.7 μm	165.2 ± 29.4 μm	122.6 ± 23.5 μm	179.8 ± 39.3 μm	75.8 ± 9.5 μm
NCR							2.4	1.4

*Mean and standard deviation for Cell area (CA); Maximum cell diameter (L, length), Minimum cell diameter (W, Width), Diameter (D) for spherical cells; NCR, Nucleocytoplasmic ratio*.

### Intraindividual Analysis

Intraindividual analysis showed significant differences for TS (*P* ≤ 0.01) and RBC (*P* ≤ 0.01), although all parameters were within the established range. For both parameters, values were higher when individuals were juvenile (Supplementary Table 2).

### Correlations

**Figure 3:**
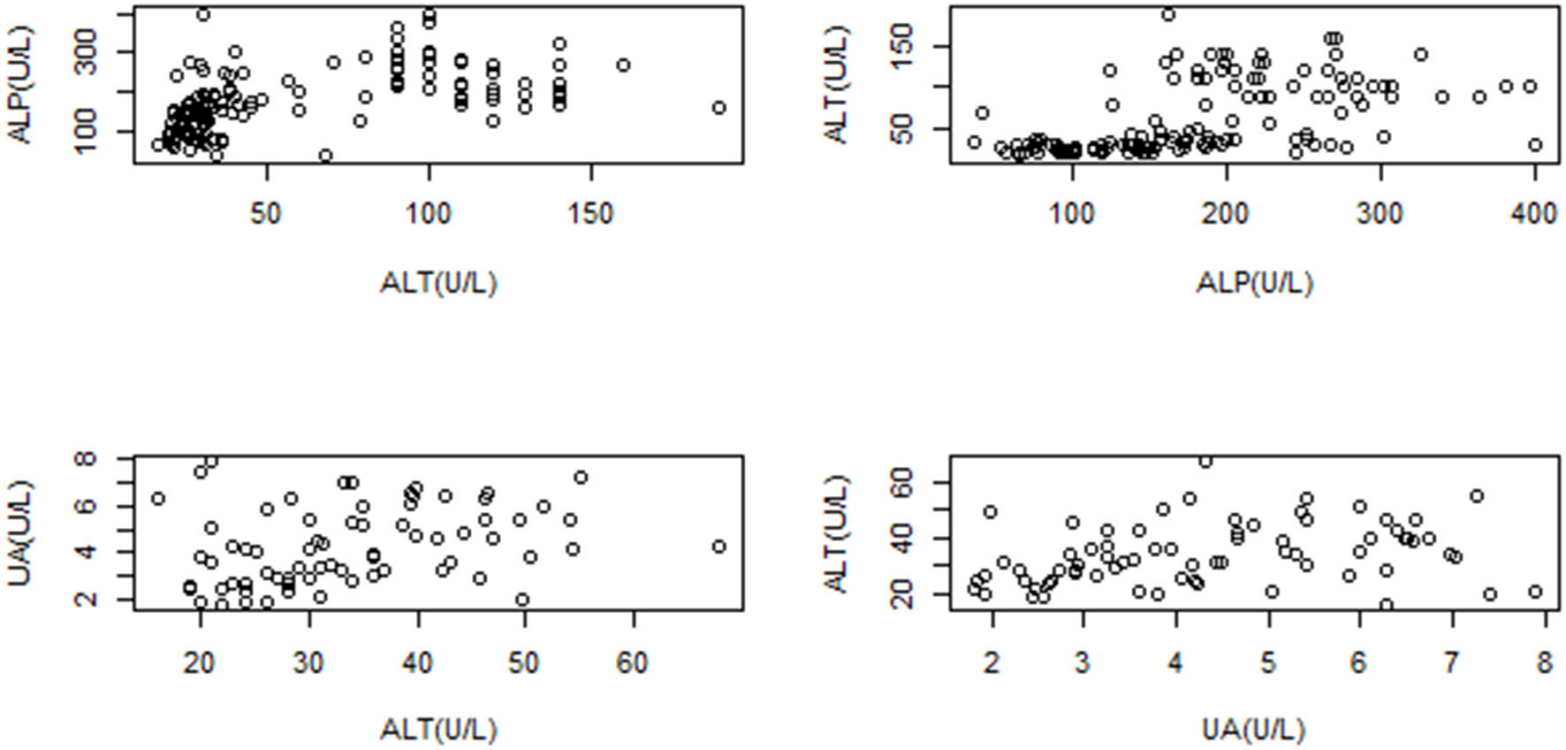
Dispersion correlation displayed for ALT-ALP (*P* ≤ 0.05, rho = 0.6353); and ALT-UA (*P* ≤ 0.01, rho = 0.4716) of *Crocodylus intermedius*, showing moderate correlation.

Moderate correlations were found between ALT–ALP (*P* ≤ 0.01, rho = 0.63) and ALT–UA (*P* ≤ 0.01, rho = 0.47; Figure 3). No lineal model could explain the hematological or biochemical variables as a function of bodyweight or total length.

## Discussion

This study represents the most extensive sampling of a captive population of *C. intermedius*. Here, we report baseline data for hematology, serum biochemistry, and blood cell morphometrics.

The *C. intermedius*' WBC showed similar ranges to captive individuals of *Crocodylus niloticus* and *Crocodylus moreletti*; however, the values were lower than those observed in wild individuals of the same species (Supplementary Table 4). Lower frequency of pathogen exposure and aggressions, as product of controlled settings, and reduced competition and then seasonal induced stress could be determinant factors for the lower WBC in captive crocodiles; in contrast, turtles and other reptiles generate higher leukocyte counts secondary to captivity stress (19, 36, 37). Heterophils were the most frequent leukocyte, agreeing with the Manzanilla et al. (11). However, this differed from other crocodilians with predominant lymphocyte leukograms (ranging up to 80%; Supplementary Table 4). Species-specific understanding of the baseline data of diagnostic tools is essential for posterior applicability (38). Based on this, the relationship between maintenance conditions and the leukogram spectrum among crocodilians must be studied to clarify this species' actual findings.

Higher RBC, PCV, HGB, and MCHC concentrations of females recall *C. niloticus* and *C. moreletti* findings, whereas the higher WBC observed in females differs from those species and *Crocodylus palustris*, where males show slight to markedly higher values [(39); Supplementary Tables 1, 4]. Intraindividual analyses showed significant differences in TS, RBC, and PCV; higher values were found in juveniles. Statistical differences for the same parameters were observed on the general sample, but higher values corresponded to adults. Previous reports of C. *niloticus* show significant differences for PCV, HGB, total protein, globulins, and AST concentrations according to the age category; likewise, higher values correspond to adult samples (13). According to this, we determined that certain analytes changed during the covered period for crocodiles, as it has been reported for other reptiles (40). Furthermore, we also recognized an absence of strict linearity for age-related analytes' shifts. Actually, one must consider that changes across time could be influenced by other intrinsic and/or extrinsic factors (41). Further studies focusing on intraindividual analyzes are desirable and should give an overview of analytes' behavior over time.

They normally proposed ALT values for reptiles are lower or equal to 20 U/L, whereas previous reports for the genus *Crocodylus* sp. document upper limits near 60 U/L (13). However, our results showed an upper limit of 100 U/L. Although ALT is a non-organ-specific enzyme, high values should not be taken as indicators of hepatic pathology by themselves; neither can they be strictly associated with specific pathologies without further clinical information and analyses (23). UA values varied across upper and lower limits against the previous reports for *Crocodylus* sp. (Supplementary Table 4). Feeding habits and husbandry conditions influence both ALT and UA and other analytes. Further studies might consider including wild and captive populations to determine how seasonality, feeding frequencies, quality, and variety of diet interact with biochemistries or hematology in the species.

Some biochemistries by themselves might not be tissue-specific on normal or pathological conditions, as we mentioned. However, when correlated analyses are carried out, a concerted behavior among analytes could provide more information about a target tissue (e.g., ALT with ALP or UA; could offer evidence about either liver or kidney tissue, respectively). Then, we proposed a correlation analysis between biochemistries as a possibility for a wider comprehension of available analytes, whose primary utility is still not clearly elucidated on reptile clinical pathology. This could be considered preliminary data for further controlled and extensive studies to understand their clinical applicability.

Sex-related differences for biochemistries such as CK, ALP, and CREA have not been reported previously; either way, formerly cited factors, and additional ones, such as the amount of displacement and enclosure type/size, could be related to higher values on females and/or additional variations.

Our limited body morphometric data (sub-adult sample) could be the reason for absent correlations between body morphometry and hematological or biochemical values in the current study. In contrast, Manzanilla et al. (11) report negative correlations for total length vs. HGB and total length vs. WBC; nevertheless, Scheelings et al. (42) find no correlations.

Peripheral blood cell morphology was akin to that reported for crocodilians and other reptile species (24–26, 43). Usually, polychromatophils are described as similar-sized or smaller than mature erythrocytes for birds and reptiles (27, 28). However, an inverse size proportion for *C. intermedius* was observed (Table 4). Azurophil-like cells were infrequently seen. Colorless and large vacuoles were observed in one individual's thrombocytes. Clear vacuoles have been described in lizard thrombocytes as glycogen storage with positive staining with Schiff's periodic acid (29). Also, *Progarnia* infection is described as thrombocyte-related parasites in crocodilians, causing cell morphological variations (30). However, we did not see chromatin nor other parasitic-like morphology. Supplementary techniques must be performed to clarify this isolated finding. We endorse cytochemical analysis to distinguish these and other unclassified cells and for elucidating morphological alterations.

This is the first study of hematology, biochemistry, and blood cell morphometrics of Colombian populations of *C. intermedius*. Intraindividual analyses could offer some valuable physiological data and deserves further studies. Sex- and age-based variations for both hematological and biochemistries on the species must be considered for routine evaluations. By preference, biochemical parameters and their correlations should be in-depth studied for assumptions about their diagnostic value among crocodiles and reptile species (38). Displayed data provide baseline information for health assessment. Likely, due to the influence of environmental, diet, husbandry, and stress factors, the reference intervals obtained were wide, as it is frequently observed in reptiles (16, 25). This work contributes as a useful tool for veterinary health assessment in reproduction and reintroduction programs for the species.

## Data Availability Statement

The raw data supporting the conclusions of this article will be made available by the authors, without undue reservation.

## Ethics Statement

The animal study was reviewed and approved by Science Faculty Ethics Committee, Universidad Nacional de Colombia.

## Author Contributions

Manuscript writing and editing, statistical analyses, and graphics and tables assembling developed by SB-V and CR-A. Statistical design and database arrangement accomplished by ZR-S and SB-V. Project scheme in charge of NM and CM-T. Literature compilation, morphological cells studies, and microphotographs developed by SB-V. Second sampling phase (2019-2020, sampling, processing, storage, and database transcription) was carried out by CR-A and CM-T. CM-T collected first phase samples (2010-2013) and offered the dataset. All authors contributed to the article and approved the submitted version.

## Conflict of Interest

The authors declare that the research was conducted in the absence of any commercial or financial relationships that could be construed as a potential conflict of interest.

## Publisher's Note

All claims expressed in this article are solely those of the authors and do not necessarily represent those of their affiliated organizations, or those of the publisher, the editors and the reviewers. Any product that may be evaluated in this article, or claim that may be made by its manufacturer, is not guaranteed or endorsed by the publisher.

## Abbreviations

ALT: Alanine aminotransferase
ALP: Alkaline phosphatase
UA: Uric acid
RBC: Red blood cell count
WBC: White blood cell count
PCV: Packed cell volume
HGB: Hemoglobin
MCHC: Mean corpuscular hemoglobin concentration
TS: Total solids
AST: Aspartate aminotransferase
CREA: Creatinine
CK: Creatine kinase
LDH: Lactate dehydrogenase
RI: Reference interval.
