# Supplementary material for: Hematology, Biochemistry Reference Intervals, and Morphological Description of Peripheral Blood Cells for a Captive Population of Crocodylus intermedius in Colombia
Source: Front Vet Sci. 2021 Aug 26;8:694354. doi: 10.3389/fvets.2021.694354 (PMC8427611; doi:10.3389/fvets.2021.694354)
Supplement: Supplementary file 1 [file Table_1.pdf]

Supplementary Material

1 Supplementary Figures

1.1 Supplementary Figure 1

Body weight (W; kg) and total length (L; cm) linear correlation for *Crocodylus intermedius* (n=41).

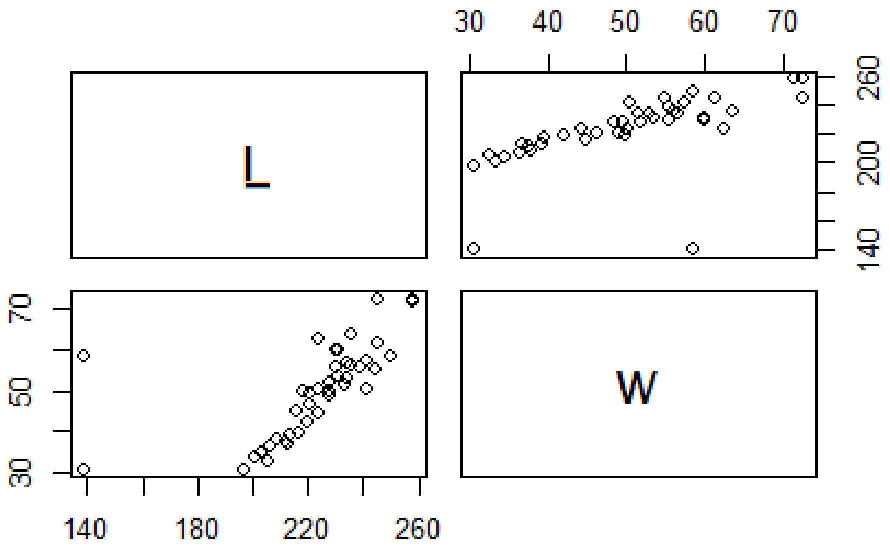

1.3 Supplementary Table 1

P-values for sex and age differences in *Crocodylus intermedius* with Mann-Whitney test. Significant differences are highlighted with bold type numbers.

Analyte	Sex	Age
	**Female vs Male**	**Adult vs Juvenile**
**PCV**	**<0,01**	0,89
**Hemoglobin**	**<0,01**	0,64
**TS**	0,05	**<0,01**
**RBC**	0,04	**0,01**
**WBC**	**0,01**	0,44
**MCV**	0,13	0,25
**MCHC**	**0,04**	-
**ALB**	0,09	-
**ALT**	0,38	0,30
**AST**	0,40	0,23
**ALP**	**0,02**	0,18
**UA**	0,24	0,84
**CK**	**0,02**	0,19
**LACT**	0,84	-
**CREA**	**0,01**	0,44

Spaces without numeric values had insufficient data for comparison. PCV: Packed cell volume. TS: Total solids. RBC: Red blood cells count. WBC: White blood cells count. MCV: Mean corpuscular volume. MCHC: Mean corpuscular hemoglobin concentration. ALB: Albumin. ALT: Alanine

aminotransferase. AST: Aspartate aminotransferase. ALP: Alkaline phosphatase. UA: Uric acid. CK: Creatine kinase. LACT: Lactate. CREA: Creatinine.

1.4 Supplementary Table 2

P-values for intraindividual comparison of analytes for eight *Crocodylus intermedius* sampled-resampled with Wilcoxon or T-test. Significant differences as bold type letter.

Analyte	Normality (Shapiro-Wilk)	P-value
**PCV**	0,01	**0,04**
**TS**	0,25	**<0,01**
**RBC**	0,43	**<0,01**
**WBC**	0,04	0,64
**MCV**	0,36	0,55
**H**	0,06	0,22
**L**	0,54	0,49
**E**	0,41	0,48
**M**	0,15	0,08
**B**	0,69	0,19
**Abs H**	0,21	0,44
**Abs L**	0,11	0,58
**Abs E**	0,08	0,33
**Abs M**	0,09	0,07
**Abs B**	0,36	0,17
**ALT**	0,003	1

Abs: Absolut count of heterophils, lymphocytes, eosinophils, monocytes, and basophils. H: Heterophils. L: Lymphocytes. E: Eosinophils. M: Monocytes. B: Basophils.

1.6 Supplementary Table 3

Comparison between hematological and serum chemistry parameters between juveniles, females, and males of *Crocodylus intermedius*. Bold values indicate parameters with statistical differences according to ANOVA or Kruskal-Wallis test (p-value <0,05).

Analyte	Juvenile		Female		Male		*p*-value
	Mean	SD	Mean	SD	Mean	SD	
**PCV (%)**	26,0	4,8	26,7	3,4	**24,5**	3,1	0,00
**TS (g/dl)**	**6,2**	1,2	**7,5**	1,3	**6,7**	1,1	0,00
**Hemoglobin (g/dl)**	8,2	0,9	**8,4**	1,2	**7,7**	1,2	0,01
**RBC (10^6/µl)**	1,0	0,4	**1,2**	0,5	1,0	0,3	0,00
**WBC (10^3/µl)**	6578,4	2452,0	**7017,9**	2459,0	**6056,2**	2649,9	0,01
**MCV (fl)**	272,6	62,3	**254,0**	82,3	291,9	68,7	0,02
**MCH (pg)**	105,7	23,9	99,8	25,4	105,0	21,8	0,51
**MCHC (g/dl)**	35,5	7,7	33,5	5,6	31,5	4,3	0,10
**Heterophils (%)**	59,1	12,8	**61,4**	13,0	**53,8**	16,4	0,01
**Lymphocytes (%)**	27,7	10,6	26,9	10,7	32,7	15,2	0,12
**Eosinophils (%)**	3,2	3,0	3,5	3,4	**5,0**	3,5	0,01
**Monocytes (%)**	1,6	2,2	2,0	3,9	1,6	3,0	0,30
**Basophils (%)**	**8,4**	4,6	6,2	4,2	6,9	4,8	0,00
**Azurophils (%)**	0,0	0,0	0,0	0,2	0,0	0,3	0,46
**Heterophils (10^3/µl)**	3988,5	1963,7	**4437,5**	2014,0	**3331,7**	2042,9	0,00
**Lymphocytes (10^3/µl)**	1753,7	822,4	1800,4	869,1	1961,1	1311,1	0,90
**Eosinophils (10^3/µl)**	192,4	173,9	211,1	196,4	262,9	202,4	0,12
**Monocytes (10^3/µl)**	106,9	152,6	153,3	293,2	116,5	269,8	0,28
**Basophils (10^3/µl)**	**547,5**	345,3	394,2	251,3	381,3	280,2	0,00
**Azurophils (10^3/µl)**	0,0	0,0	2,3	21,8	2,8	18,2	0,47
**Glucose (mg/dl)**	92,0	-	130,3	23,0	122,2	24,4	0,17
**ALB (g/dl)**	1,8	0,4	**2,1**	0,3	2,0	0,3	0,01
**Globulin (g/dl)**	**6,1**	1,4	**6,4**	1,6	**5,2**	1,6	0,00
**ALT (U/l)**	40,2	25,1	56,0	36,4	55,0	44,7	0,25
**AST (U/l)**	130,4	89,0	106,8	67,0	90,0	52,9	0,53
**ALP (U/l)**	169,3	84,4	192,4	85,8	152,7	49,7	0,06
**CHOL (mg/dl)**	**248,6**	47,3	255,9	38,4	**282,9**	53,5	0,03
**UA (mg/dl)**	4,3	1,0	4,6	1,8	3,7	1,6	0,38

**CREA (mg/dl)**	0,5	0,1	**0,5**	0,1	0,5	0,1	0,01
**CK (U/l)**	3436,0	2343,4	2551,2	2157,2	**1564,2**	1224,0	0,03
**LACT (mg/dl)**	102,2	-	65,0	36,1	65,2	49,3	0,73
**LDH (U/l)**	-	-	25,5	13,2	19,1	10,2	0,25

1.8 Table 4

Comparison of hematological and serum chemistry values between *Crocodylus intermedius* analyzed in this study and other crocodilians of the same genus, studies done either on captivity or in wildlife.

	This study (*Crocodylus intermedius*, n= 326)		*C. intermedius* (Juv) Wild (n= 80) [a]		*C. johnstoni* Wild (n= 39) [b]		*C. porosus* Captive (n= 39) [c]		*C. palustris* Captivity (n= 16) [d]	
**Analyte**	**Mean ± SD**	**Min-Max**	**Mean ± SD**	**Min-Max**	**Mean ± SD**	**Min-Max**	**Mean**	**Min-Max**	**Mean ± SD**	**Min-Max**
**PCV (%)**	26,3 ± 3,7	17 - 36	24,7 ± 2,5	-	23,1 ± 3,4	18,0 - 32,0	-	17 - 41	22,4 ± 4,0	17 - 29
**TP/TS (g/dl)**	7,0 ± 1,4	3,9 - 10,2	-	-	3,96 ± 1,4	1,5 - 7,0	-	4,1 - 7,0	-	-
**Hemoglobin (g/dl)**	8,2 ± 1,2	5,1 - 12,1	8,27 ± 1,6	-	-	-	-	4,7 - 12,2	7,55 ± 1,34	5,2 - 9,7
**RBC (10^6/µl)**	1,1 ± 0,4	0,47 - 2,28	-	-	-	-	-	0,6 - 1,3	0,71 ± 0,14	0,48 - 0,95
**WBC (10^3/µl)**	6,8 ± 2,5	2,2 - 15,1	6,60 ± 1,0	-	8,0 ± 3,9	2,2 - 17,5	-	6,4 - 25,7	-	-
**MCV (fl)**	263,0 ± 76,7	100 - 592	-	-	-	-	-	240 - 311	323,6 ± 68,3	232 - 492
**MCH (pg)**	10,1 ± 2,5	5,7 - 17,4	-	-	-	-	-	72 - 92	108,6 ± 22,0	78,8 - 163
**MCHC (g/dl)**	33,1 ± 5,4	22,1 - 50,4	-	-	-	-	-	261 - 319	33,6 ± 0,44	33,0 - 34,1
**Heterophils (%)**	60,8 ± 12,1	20 - 83	55,8 ± 8,8	-	-	-	-	-	-	-
**Heterophils (10^3/µl)**	4,10 ± 1,94	0,3 - 9,5	-	-	1,3 ± 1,3	0,1 - 6,8	-	0,8 - 7,4	5,15 ± 1,71	3,33 - 9,72
**Lymphocytes (%)**	27,2 ± 10,5	6 - 65	31,8 ± 10,2	-	-	-	-	-	-	-
**Lymphocytes (10^3/µl)**	1,72 ± 0,74	0,3 - 3,6	-	-	5,1 ± 2,5	1,4 - 9,8	-	4,5 - 21,6	3,01 ± 1,11	1,2 - 4,93
**Eosinophils (%)**	3,6 ± 3,3	0 - 16	8,3 ± 5,6	-	-	-	-	-	-	-
**Eosinophils (10^3/µl)**	0,18 ± 0,15	0 - 0,62	-	-	0,7 ± 0,5	0,0 - 1,6	-	0,0 - 0,7	0,33 ± 0,17	0,15 - 0,6
**Monocytes (%)**	1,7 ± 3,4	0 - 20	1,6 ± 1,9	-	-	-	-	-	-	-
**Monocytes (10^3/µl)**	0,05 ± 0,085	0 - 0,34	-	-	1,0 ± 0,8	0,0 - 2,7	-	0,0 - 1,2	0,09 ± 0,08	0 - 0,26
**Basophils (%)**	6,6 ± 4,2	0 - 20	3,0 ± 2,1	-	-	-	-	-	-	-

**Basophils (10^3/µl)**	0,40 ± 0,24	0 - 1,09	-	-	0,0 ± 0,1	0,0 - 0,6	-	0,0 - 0,7	0,03 ± 0,07	0 - 0,17
**Azurophils [a] (%)**	0,0 ± 0,2	0 - 3	-	-	-	-	-	-	-	-
**Azurophils [a] (10^3/µl)**	0,001 ± 0,001	0 - 0,28	-	-	-	-	-	-	-	-

Continuation

	**This study (*Crocodylus intermedius*, n= 326)**		***C. intermedius* (Juv) Wild (n= 80) [a]**		***C. johnstoni* Wild (n= 39) [b]**		***C. porosus* Captive (n= 39) [c]**		***C. palustris* Captive (n= 16) [e]**	
**Analyte**	**Mean ± SD**	**Min-Max**	**Mean ± SD**	**Min-Max**	**Mean ± SD**	**Mean ± SD**	**Min-Max**	**Mean ± SD**	**Min-Max**	**Mean ± SD**
**ALP (U/l)**	181,1 ± 79,5	37 - 400	-	-	-	-	-	31 - 180	44,7 ± 10,3	30 - 66
**CHOL (mg/dl)**	253,5 ± 40,3	164 - 357	-	-	-	-	-	42,5 - 278	236,7 ± 21,9	192 - 262
**UA (mg/dl)**	4,4 ± 1,7	1,8 - 7,9	-	-	1,1 ± 0,42	0,4 - 1,89	-	2,8 - 16,6	3,86 ± 1,3	2,4 - 7,0
**CREA (mg/dl)**	0,5 ± 0,1	0,4 - 0,7	-	-	-	-	-	0,22 - 0,57	0,43 ± 0,11	0,3 - 0,6
**CK (U/l)**	1825 ± 1288	187 - 5915	-	-	1086 ±1243	109 - 4448	-	-	-	-
**LACT (mg/dl)**	66,2 ± 41,8	16 - 178	-	-	-	-	-	-	-	-
**LDH[b] (U/l)**	21,5 ± 10,4	4 - 47,1	-	-	-	-	-	-	-	-
**Glucose (mg/dl)**	128,1 ± 23,5	92 - 186	-	-	64,8 ± 18	36,0 - 111	-	81,0 - 217	58,3 ± 7,23	50 - 74
**ALB (g/dl)**	2,0 ± 0,3	1,4 - 3	-	-	0,77 ± 0,3	0,0 - 1,5	-	1,4 - 2,3	1,11 ± 0,14	0,9 - 1,3
**ALT (U/l)**	40,6 ± 21,1	11,5 - 100	-	-	-	-	-	11 - 51	57,7 ± 14,0	44 - 97
**AST (U/l)**	103,3 ± 64,6	25 - 260	-	-	36,5 ± 12,8	19,0 - 74,0	-	23 - 157	50,9 ± 9,12	30 - 65

[a] Wild juvenile *Crocodylus intermedius*, (Manzanilla et al 2011, 360–356)

[b] Wild *Crocodylus johnstoni* (Scheelings et al 2016, 959–961)

[c] Captive *Crocodylus porosus* (Millan et al 1997, 814–817)

[d] Captive *Crocodylus palustris* (Stacy and Whitaker 2000, 339–347)

Continuation

	**This study (*Crocodylus intermedius*, n= 326)**		***C. niloticus* Wild (n= 38) [e]**		***C. niloticus* Captive (n= 44) [e]**		***C. moreletti* Captive (n= 47) [f]**		***C. moreletti* Wild (n= 45) [f]**	
**Analyte**	**Mean ± SD**	**Min-Max**	**Mean**	**Min-Max**	**Mean**	**Min-Max**	**Mean**	**Min-Max**	**Mean**	**Min-Max**
**PCV (%)**	26,3 ± 3,7	17 - 36	17,9	14 - 22	27,2	24 - 31	24,5	23,2 - 25,7	24,6	22,7 - 26,4
**TP/TS (g/dl)**	7,0 ± 1,4	3,9 - 10,2	4,12	2,8 - 5,7	5,3	-	-	-	-	-
**Hemoglobin (g/dl)**	8,2 ± 1,2	5,1 - 12,1	7,1	4,7 - 9,5	8,7	7,8 - 9,5	-	-	-	-
**RBC (10^6/µl)**	1,1 ± 0,4	0,47 - 2,28	0,59	0,35 - 1	0,92	0,6 - 1,31	1,1	1,02 - 1,17	1,04	0,92 - 1,16
**WBC (10^3/µl)**	6,8 ± 2,5	2,2 - 15,1	11,28	3,75 - 26,22	6,4	4,0 - 11,5	8,3	8,0 - 8,5	9,9	9,4 - 10,4
**MCV (fl)**	263,0 ± 76,7	100 - 592	312,2	200 - 465	306,7	206 – 440	-	-	-	-
**MCH (pg)**	10,1 ± 2,5	5,7 - 17,4	123,2	83,8 - 220,9	97,9	65,3 - 153	-	-	-	-
**MCHC (g/dl)**	33,1 ± 5,4	22,1 - 50,4	39,6	29,0 - 47,5	31,9	29,0 - 38,3	-	-	-	-
**Heterophils (%)**	60,8 ± 12,1	20 - 83	20,5	4 - 39	13,4	6 - 20	-	-	-	-
**Heterophils (10^3/µl)**	4,10 ± 1,94	0,3 - 9,5	2,09	0,45 - 3,66	-	-	2,27	2,21 - 2,42	2,96	2,68 - 3,24
**Lymphocytes (%)**	27,2 ± 10,5	6 - 65	62	44 - 85	82,2	73 - 95	-	-	-	-
**Lymphocytes (10^3/µl)**	1,72 ± 0,74	0,3 - 3,6	7,20	1,65 - 17,83	-	-	3,93	3,73 - 4,14	4,33	4,06 - 4,59
**Eosinophils (%)**	3,6 ± 3,3	0 - 16	4,9	0 - 17	4,4	2 - 8	-	-	-	-
**Eosinophils (10^3/µl)**	0,18 ± 0,15	0 - 0,62	0,53	0 - 2,14	-	-	0,26	0,21 - 0,32	0,26	0,21 - 0,33
**Monocytes (%)**	1,7 ± 3,4	0 - 20	0,9	0 - 10	2,5	1 - 7	-	-	-	-
**Monocytes (10^3/µl)**	0,05 ± 0,085	0 - 0,34	0,09	0 - 0,79	-	-	0,08	0,05 - 0,11	0,28	0,22 - 0,33
**Basophils (%)**	6,6 ± 4,2	0 - 20	5,9	0 - 16	-	-	-	-	-	-
**Basophils (10^3/µl)**	0,40 ± 0,24	0 - 1,09	0,69	0 - 2,9	-	-	1,5	1,44 - 1,72	1,80	1,62 - 1,92
**Azurophils [a] (%)**	0,0 ± 0,2	0 - 3	5,1	0 - 21	-	-	-	-	-	-
**Azurophils [a] (10^3/µl)**	0,001 ± 0,001	0 - 0,28	0,6	0 - 3,93	-	-	0,15	0,11 - 0,18	0,26	0,19 - 0,33

Continuation

	This study (*Crocodylus intermedius*, n= 326)		*C. niloticus* Wild (n= 38) [e]		*C. niloticus* Captive (n= 44) [e]		*C. moreletti* Captive (n= 47) [f]		*C. moreletti* Wild (n= 45) [f]	
**Analyte**	**Mean ± SD**	**Min-Max**	**Mean**	**Min-Max**	**Mean**	**Min-Max**	**Mean**	**Min-Max**	**Mean**	**Min-Max**
**ALP (U/l)**	181,1 ± 79,5	37 - 400	21,1	3 - 72	-	-	-	-	-	-
**CHOL (mg/dl)**	253,5 ± 40,3	164 - 357	212,2	0 - 381,2	-	-	283	249 - 316	228,7	196 - 260
**UA (mg/dl)**	4,4 ± 1,7	1,8 - 7,9	2,0	0,67 - 5,04	4,03	-	1,95	1,51 - 2,33	4,48	3,13 - 5,84
**CREA (mg/dl)**	0,5 ± 0,1	0,4 - 0,7	0,38	0,19 - 0,63	-	-	2,74	2,44 - 3,04	2,76	2,22 - 3,3
**CK (U/l)**	1825 ± 1288	187 - 5915	-	-	-	-	-	-	-	-
**LACT (mg/dl)**	66,2 ± 41,8	16 - 178	-	-	-	-	-	-	-	-
**LDH[b] (U/l)**	21,5 ± 10,4	4 - 47,1	-	-	-	-	-	-	-	-
**Glucose (mg/dl)**	128,1 ± 23,5	92 - 186	68,4	32,4 - 86,4	82,3	-	64,3	58,9 - 69,7	77,7	67,4 - 88,1
**ALB (g/dl)**	2,0 ± 0,3	1,4 - 3	1,4	1,1 - 1,9	1,9	-	-	-	-	-
**ALT (U/l)**	40,6 ± 21,1	11,5 - 100	43,9	15 - 63	13,1	-	17,8	13,1 - 22,5	20,2	15,0 - 25,4
**AST (U/l)**	103,3 ± 64,6	25 - 260	66,5	14 - 211	16,6	-	-	-	-	-

[e] Wild and captive *Crocodylus niloticus*, (Lovely et al 2007, 137–144)

[f] Wild and captive *Crocodylus moreletti* (Padilla et al 2011, 511–522)
